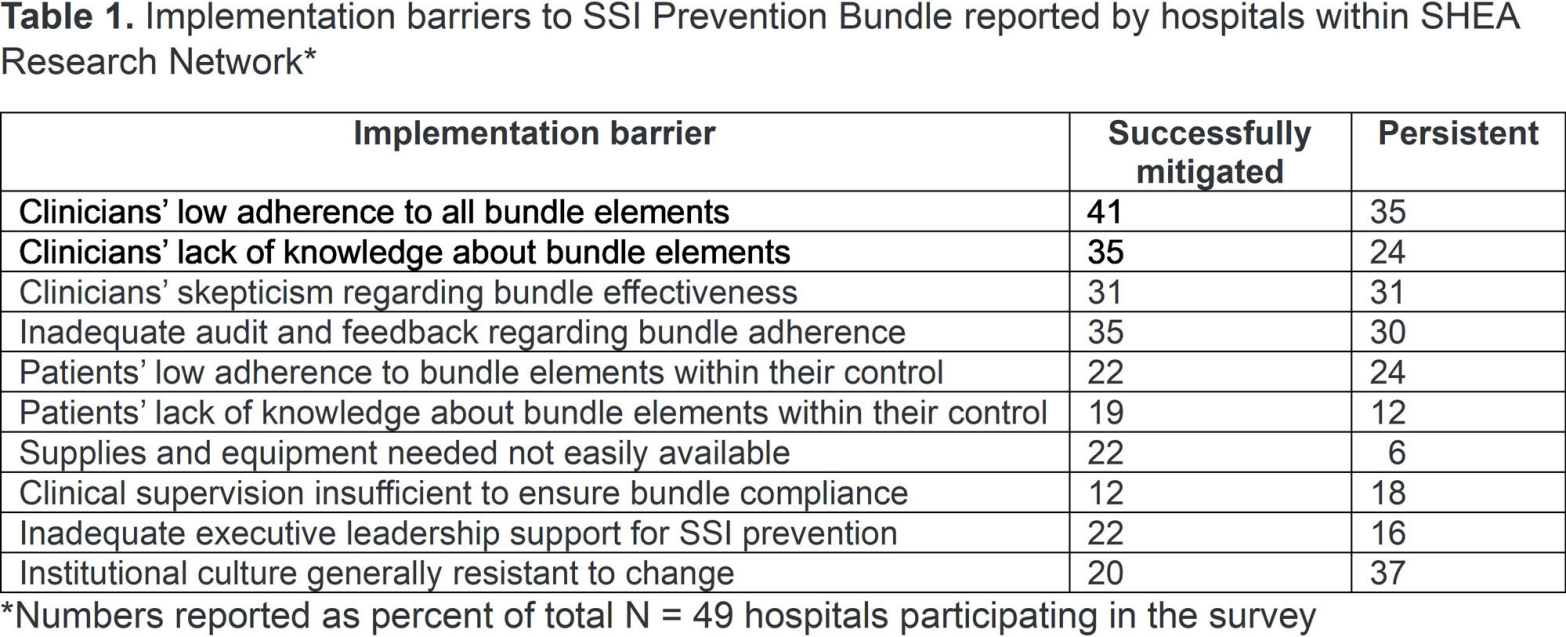# Bundle Implementation to Prevent Surgical Site Infections - A Study of SRN Hospitals

**DOI:** 10.1017/ash.2024.318

**Published:** 2024-09-16

**Authors:** Aurora Pop-Vicas, Michelle Zimbric, Michelle Schmitz, Gabrielle Hatas, Nasia Safdar

**Affiliations:** University of Wisconsin School of Medcine and Public Health; Infecitous Disease, Department of Medicine, School of Medicine and Public Health; UW Health - University Hospital; University of Wisconsin Hospital and Clinics

## Abstract

**Background:** Guidelines recommend bundles with multiple infection control elements to prevent surgical site infections (SSI). Although effective in multiple research studies, little is known about the implementation of such complex bundles in the real-world clinical setting. **Methods:** A survey was distributed to the SHEA Research Network (SRN) hospitals during November 2022 – December 2023, to assess processes related to the implementation of SSI prevention bundles in colorectal surgery. **Results:** Of the 93 US and international hospitals within SRN, 49 completed the survey (53% response rate). The mean volume of colorectal surgeries per year was 377 (median 400). Figure 1 shows the individual elements of SSI prevention bundle reported as consistently used in most surgeries. There were no significant differences between hospitals with high vs. low volume (cut-off 400 surgeries), except for wound protectors or retractors, more likely to be used in high-volume hospitals (P = 0.047). A formal process for auditing adherence was reported by 71% of respondents for antibiotic prophylaxis, and 51% for skin prep, with the remaining elements audited < 50% of the time. Feedback of audited adherence to surgeons occurred < 50% of the time for all bundle elements, except antibiotic prophylaxis (59%). Table 1 shows the most common barriers reported as either successfully mitigated or still persistent at the time of the survey. High-volume hospitals were more likely to report persistent clinicians’ low bundle adherence (P = 0.016) and inadequate bundle adherence audit and feedback (P = 0.0016). **Conclusion:** Implementation of guideline-recommended colorectal SSI Prevention bundles remains highly variable. Further research aiming to develop strategies that optimize implementation and adherence is needed.

**Figure:**
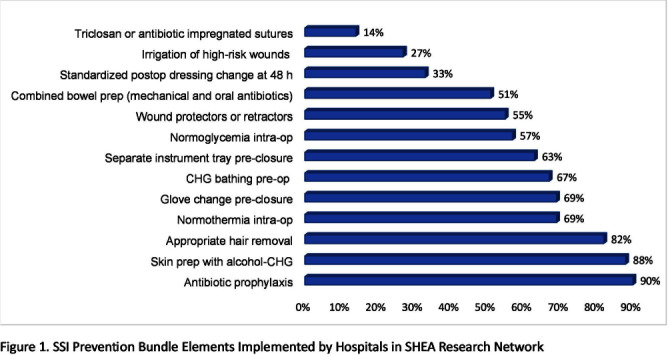

**Figure:**